# Cholesterol goals, statin use and residual cardiovascular risk estimated by SMART score: Study of a Nicaraguan population

**DOI:** 10.1016/j.ijcrp.2023.200192

**Published:** 2023-06-23

**Authors:** Ginner Odorico Rizo Rivera, Marion Jose Valladares, Hildebrando M. Toledo Vargas, Elibeth Chavez, Alejandro A. Garcia de la Rocha, Luis A. Urcuyo Hernandez, Jose Daniel Meneses Mercado

**Affiliations:** a CardioCenter Clinic, Jinotega-Matagalpa, Nicaragua; b Ministerio de Salud, Nicaragua; c National Autonomous University of Nicaragua-Leon, Nicaragua; d UNAN-Managua, Centro Regional de Especialidades Médicas de Matagalpa, Nicaragua; e National Autonomous University of Nicaragua, Nicaragua; f Dr. Serafín Ruiz de Zárate Ruiz, Villa Clara, Cardiocentro Ernesto Che Guevara, Santa Clara, Cuba; g UNAN-Managua, San José Clinic, Granada, Nicaragua; h UNAN-Managua, Clínica de Diabetes y Pie Diabético, Estelí, Nicaragua; i Interventional Cardiology, Clínica del Valle, Managua, Nicaragua

**Keywords:** Residual cardiovascular risk, SMART risk score, Statins

## Abstract

**Introduction:**

Adverse cardiovascular events that arise in patients with established cardiovascular disease have prompted researchers to seek variables that can help estimate residual cardiovascular risk and aid in its reduction. In Latin-America, there is limited data assessing this type of risk.

**Objective:**

Estimate residual cardiovascular risk in ambulatory patients diagnosed with Chronic Coronary Syndrome (CCS) using the SMART-Score scale seen at five clinics in Nicaragua; determine the prevalence of patients that achieve a serum LDL level of <55 mg/dL; and describe the use of statins in these patients.

**Methods:**

A total of 145 participants previously diagnosed with CCS seen regularly in ambulatory visits were enrolled. A survey was completed, including epidemiological variables that allowed the calculation of a SMART score. Data analysis was conducted using SPSS version 21.0.

**Results:**

A 46.2% of participants were male, the average age was 68.7 years (11.4 SD), 91% had hypertension, 80.7% had a BMI ≥25. Under the SMART Score risk classification per Dorresteijn et al. the following risk distribution was found: 2.8% low, 31% moderate, 20% high, 13.1% very high and 33.1% extremely high. Per the risk classification of Kaasenbrood et al., 2.8% were in the 0–9% group, 31% in the 10–19%, 20% in 20–29% and 46.2% were in the ≥30% group. A 64.8% did not meet LDL goals.

**Conclusion:**

There is an inadequate control of cLDL levels in patients with CCS, and the appropriate available therapeutic resources aren't being utilized. It is important to achieve a proper control of lipid levels in order to improve cardiovascular outcomes, despite currently being far from these goals.

## 1 Introduction

The Framingham Study coined the term “risk factor”, this is defined as an element or measurable characteristic with causal relation, that constitutes an independent predictive factor and significant risk of developing the disease of interest. Prevention of cardiovascular diseases is an important element of public health policy, at different levels of healthcare, due to different reasons and sustained by the control of risk factors [1,2].

The estimation of cardiovascular risk may be done in an opportunistic or systematic fashion. The opportunistic screening is done without predefined strategies, but is conducted once the opportunity for it arises. Systematic screening can be conducted in the general population as part of a screening program or in concrete subpopulations of interest, such as people with family history of premature cardiovascular disease (CVD) [3].

Many scoring systems to assess for risk of CVD have been developed, including: Framingham, Assign, Q-Risk, Procam, Coure amongst others. The 2008 Framingham score is a worldwide reference and is used in various Latin-American countries as the foundation for primary prevention; this score predicts death due to coronary disease, heart failure, stroke, intermittent claudication and peripheral vascular disease [4].

A recent revision in concepts made by the European Society of Cardiology (ESC) in 2019 regarding Coronary Artery Disease (CAD), emphasizes that given the different possible clinical presentations of CAD, it can be categorized as either Acute Coronary Syndrome (ACS) or Chronic Coronary Syndrome (CCS). Furthermore, the ESC defines CCS as “the different evolutionary phases of CAD, excluding situations in which an acute coronary artery thrombosis dominates the clinical presentation” [5].

Recently, efforts have been made to study the likelihood of new adverse cardiovascular events occurring in patients who have already suffered an acute cardiovascular episode. The risk of developing new events in these patients has been termed “Residual Risk” [[6], [7], [8]].

A holistic definition for Residual Cardiovascular Risk (RCVR) states that it is the residual risk due to the progression of established vascular damage that persists in patients treated with current evidence based standard of care, including the treatment of established risk factors such as dyslipidemia, arterial hypertension, hyperglycemia, and unhealthy lifestyles; as well as the risk related to the development of newer risk factors [6].

Several non-lipid factors [i.e. age, sex, smoking, alcohol intake, sedentarism, Diabetes Mellitus, obesity, hypertension] and lipid factors (i.e. high levels of c-LDL, triglycerides, and low levels of c-HDL) contribute to cardiovascular risk. Even though statin therapy along with the optimization of lifestyles may successfully decrease c-LDL and reduce the rate of cardiovascular events in many patients, those with persistent lipidic anomalies may still experience such events as they do not achieve a reduction of RCVR [7].

Two of the validated scoring systems for estimating RCVR most commonly used are the SMART (Secondary Manifestations of ARTerial disease) score and the TIMI Risk Score [[8], [9], [10]].

Given that there is a scarcity of studies on estimation of RCVR in the Nicaraguan population of patients with chronic coronary disease, this study aims to evaluate the residual cardiovascular risk in patients with chronic coronary syndrome who are seen in an outpatient setting.

### 1.1 General objective

To evaluate the residual cardiovascular risk in outpatients diagnosed with Chronic Coronary Syndrome.

### 1.2 Specific objectives

To stratify the residual cardiovascular risk in outpatients diagnosed with Chronic Coronary Syndrome using the SMART-Score risk scale.

To determine the percentage of patients with Chronic Coronary Syndrome that achieve the lipid goal of LDL below 55 mg/dL.

To describe the use of statin therapy in these patients.

#### 1.2.1 Material and methods

A cross-sectional descriptive study was conducted with 145 patients diagnosed with Chronic Coronary Syndrome who were followed up with in an ambulatory setting in five separate privately run outpatient clinics: Cardiocenter-Jinotega, Centro Regional de Especialidades Medicas-Matagalpa, Clinica de Diabetes y Pie Diabetico-Esteli and in the Clinica San Jose de Granda during the period of April 2021 to April 2022.

The following clinical features were taken into account for the diagnosis of CCS: prior episode of Acute Coronary Syndrome (ACS), clinical symptoms plus positive ECG, clinical symptoms with positive stress echocardiogram test, clinical symptoms with positive stress test, clinical symptoms with positive coronary angiography, and clinical symptoms with positive nuclear cardiology tests.

Case report forms were used to collect the following variables: sex, age, body mass index, smoking habits, history of diabetes mellitus, arterial hypertension, cerebrovascular disease, peripheral arterial disease, history of aortic aneurism disease and prior acute coronary disease, statin therapy, type of statin prescribed; physical exam: systolic blood pressure, diastolic blood pressure; lab work: total cholesterol, HDL, LDL, creatinine, GFR per CKD-EPI and glycemia. These variables were used to calculate a SMART score [[8], [9], [10], [11]].

The study questionnaire was administered by the study co-investigator physicians at their clinical practice offices during standard of care clinical consultation visits, blood pressure and other anthropomorphic data was also collected at the same visit. Blood samples were drawn after the clinic visit as required by standard of care and the study protocol.

Hypertension was defined as participants who were taking at least one medication prescribed for high blood pressure, or had a history of systolic blood pressure >140 mmHg or diastolic blood pressure >90 mmHg as recorded in medical records.

The online U-Prevent [11] calculator was used to estimate SMART-Scores taking into account the following variables: age, sex, smoking habits, blood pressure, history of diabetes, time since diagnosis of CCS, HDL, total cholesterol, GFR by CKD-EPI, and average PRC.

The residual cardiovascular risk of each participant was classified in two ways based on their respective SMART Score: according to the original study in one of 5 groups, and according to the validation study in one of 4 groups. The 5 risk groups in the original study are: low 10-year risk, moderate 10-year risk, high 10-year risk, very high 10-year risk, and extremely high 10-year risk [10]. The risk groups in the validation study are as follows: 0–9% risk, 10–19% risk, 20–29% risk, and ≥ 30% risk [8].

Statistical analyses were performed with SPSS statistical package version 21.0 for Windows. Continuous variables were summarized by their averages and standard deviations. Categorical variables are reported in absolute values and percentages. The normal distribution of variables was evaluated with the Kolmogorov Smirnov test and a threshold of significance of p = 0.05.

The study design was elaborated in accordance with the principles of the Helsinki Declaration [12], reviewed and approved by the ethics committee of each healthcare provider. Each participant received a detailed explanation of the purpose of the study and their informed consent was obtained in writing. No study interventions were conducted, and participants received the standard of care according to their clinical needs. All data were codified, all personal health identifiers were excluded from the database to preserve privacy and maintain confidentiality.

#### 1.2.2 Results

Table 1 shows the general characteristics of the study population, of which 53.8% was female, 31% were seen for the first time, and a majority had hypertension (91%), however, the average levels of SBP and DBP were within target ranges. A total of 80.7% of participants were classified as overweight or obese.

**Table 1:** General characteristics of participants.

Characteristics		
Sex	Male: 46.2%	Female: 53.8%
First encounter^a^	Yes: 31%	No: 69%
Hypertensive	Yes: 91%	No: 9%
BMI ≥25	Yes: 80.7%	No: 19.3%
Age (years)	Average: 68.7	SD: ±11.4
SBP (mmHg)	Average:128.7	SD: ±19
DBP (mmHg)	Average:72.2	SD: ±12

a First encounter refers to participants who were seen for the first time as an outpatient for follow-up at their respective clinic and study visit.

Table 2 shows the average and standard deviations of complimentary studies. As shown, the average values of LDL, triglycerides and creatinine are above target values.

**Table 2:** General characteristics of laboratory results.

	N	Minimum	Maximum	Average	SD
Cholesterol total mg/dl	145	65	363	162.85	58.437
Cholesterol LDL mg/dl	145	5	247	81.69	49.037
Cholesterol HDL mg/dl	145	20	87	44.34	10.051
Triglycerides mg/dl	145	50	450	175.07	72.969
Uric Acid mg/dl	96	2.38	55.00	5.5816	5.41773
Glycemia mg/dl	130	64	349	121.98	55.483
Creatinine mg/dl	130	0.60	6.90	1.1294	0.61

HDL: High-Density Lipoprotein, LDL: Low-Density Lipoprotein.

Table 3, Table 4 as well as graphs 3 and 4 show the distribution of participants according to their risk categories under SMART risk scores, both per the original study classification and the validation study classification. When considering the original study risk groups, the risk group with the highest percentage of participants was within the extremely high 10-year risk group, accounting for 33.1% of the study sample, followed by the those in the moderate 10-year risk group with a 31.0% (see Table 3). Among the risk groups of the validation study, the most prevalent was the ≥ 30% risk group with a 46% of the study population (see Table 4).

**Table 3:** Distribution of SMART Score Risk Groups per Dorresteijn et al. [8].

	Frequency	Percentage	Accumulated Percentage
Low Risk	4	2.8	2.8
Moderate Risk	45	31.0	33.8
High Risk	29	20.0	53.8
Very High Risk	19	13.1	66.9
Extremely High Risk	48	33.1	100.0
Total	145	100.0

**Table 4:** Distribution of SMART Score Risk Groups per Kaasenbrood et al. [7].

	Frequency	Percentage	Accumulated Percentage
0–9%	4	2.8	2.8
10–19%	45	31.0	33.8
20–29%	29	20.0	53.8
≥30%	67	46.2	100.0
Total	145	100.0	

The percentage of participants that exceeded the target LDL cholesterol level of <55 mg/dL was 64.5% (see Table 5), this despite that 72.4% of participants were on high intensity statin therapy. (see Table 6).

**Table 5:** Percentage of participants that reached LDL goal lower than 55 mg/dL.

	Frequency	Percentage	Accumulated Percentage
Yes	51	35.2	35.2
No	94	64.8	100.0
Total	145	100.0

**Table 6:** Statin therapy use.

	Frequency	Percentage	Accumulated Percentage
No statin use	16	11.0	11.0
Prior use of low intensity statins	2	1.4	12.4
Prior use of moderate intensity statins	15	10.3	22.8
Prior use of high intensity statins	105	72.4	95.2
Statins were indicated but was not taken	7	4.8	100.0
Total	145	100.0	

#### 1.2.3 Discussion

It is important to raise awareness about Residual Cardiovascular Risk in the medical community and among patients, in order to bring about the necessary measures to reduce the recurrence of atherothrombotic events, which is precisely related to Residual Cardiovascular Risk [[13], [14], [15]].

Two sets of risk groups have been characterized under the SMART Score system. The original study by Dorresteijn stratified subjects in one off five different categories as previously mentioned above. Kaasenbrood grouped the highest risk groups, Very High Risk and Extremely High Risk, into one category, those with a risk ≥ 30% [8].

Age is a variable that play an important role in most risk scoring systems for cardiovascular disease, along with other cardiovascular risk factors such as smoking, diabetes, hypertension, dyslipidemia, among others. In the original study done by Dorresteijn et al. [9], the average age of the studied population was 60 years of age in the initial cohort, and 61 years of age in the validation cohort, similar results were reported by Kaasenbrood et al. [7]. In our population, the average age was higher at 68 years among our participants with CCS.

Dorresteijn and colleagues found that in regards to sex, 74% of the initial population were males, similar to a 75% found in the validation cohort, as also seen in the Kaasenbrood et al. study [8]. The present study on the contrary to the original studies, found that females was the larger group by sex, representing 53.8% of all participants.

A 32% of participants were seen for the first time at the study clinic sites, which interestingly is similar to the percentage of patients who were not on high intensity statin therapy (see Table 1, Table 6). Moreover, only 18.2% of those who were seen for the first time had achieved their LDL goals, compared to 48.8% of those who had recurring encounters. This shows that although there seems to be a higher rate of LDL goals achieved by those who had regular clinic visits, the majority of them still had not achieved their LDL goal (see Table 7) (see Table 8).

**Table 7:** LDL Goal by First encounter^a^.

	Achieved LDL Goal							
	Yes			No			Subtotal	
	Count	R%	C%	Count	R%	C%	Count	C%
First encounter	8	18.2%	16.7%	36	81.8%	46.2%	44	34.6%
Not first encounter	40	48.8%	83.3%	42	51.2%	53.8%	82	64.6%
Total	48		100%	78		100%	126	100%

a First encounter refers to participants who were seen for the first time as an outpatient for follow-up at their respective clinic and study visit.

**Table 8:** Distribution of LDL goal by sex.

		Achieved LDL Goal						
		Yes			No			Total
		Count	Row N%	Column N %	Count	Row N %	Column N %	
Sex	Male	20	29.9%	39.2%	47	70.1%	50.0%	100%
	Female	31	39.7%	60.8%	47	60.3%	50.0%	100%
Total		51		100%	94		100%

**Table 9:** LDL Goal by SMART Score Risk Groups per Dorresteijn et al.

	Achieved LDL Goal							
	Yes			No			Subtotal	
	Count	R%	C%	Count	R%	C%	Count	C%
Low Risk	3	75.0%	5.9%	1	25.0%	1.1%	4	2.8%
Moderate Risk	14	31.1%	27.5%	31	68.9%	33.0%	45	31.0%
High Risk	11	37.9%	21.6%	18	62.1%	19.1%	29	20.0%
Very High Risk	6	31.6%	11.8%	13	68.4%	13.8%	19	13.1%
Extremely High Risk	17	35.4%	33.3%	31	64.6%	33.0%	48	33.1%
Total	51		100%	94		100%		100%

**Table 10:** LDL Goal by SMART Score Risk Groups per Kaasenbrood et al. [7].

	Achieved LDL Goal							
	Yes			No			Subtotal	
	Count	R%	C%	Count	R%	C%	Count	C%
0–9%	3	75.0%	5.9%	1	25.0%	1.1%	4	2.8%
10–19%	14	31.1%	27.5%	31	68.9%	33.0%	45	31.0%
20–29%	11	37.9%	21.6%	18	62.1%	19.1%	29	20.0%
≥30%	23	34.3%	45.1%	44	65.7%	46.8%	67	46.2%
Total	51		100%	94		100%		100%

In relation to hypertension as a risk factor for cardiovascular disease, there is known to be a direct proportional correlation between higher levels of SBP and DBP with mortality due to coronary and cerebrovascular disease, in some populations, the relative risk has been estimated at an OR of 1.5 [16]. Additionally, it has been established that there is a high prevalence of obesity in patients with established cardiovascular disease [17,18].

A high proportion of participants in our study had hypertension (91%) and were overweight or had obesity (80.7%). These percentages demonstrate the relation between these two cardiovascular risk factors and CCS and its high prevalence in the Nicaraguan population as described in prior studies [[13], [14], [19]]. The average SBP in the original study was higher at 139 mmHg^9^, than that found in the present study at 128 mmHg, as well as the average DBP, being 81 mmHg in the original study [9] as opposed to 72 mmHg in the present study.

In determining residual cardiovascular risk by the SMART score, we found a predominance of patients classified in the extremely high risk group at 33% according to the original classification of Dorresteijn (Table 3). When adding the proportions of those belonging to the extremely high, very high and high risk groups, we find a proportion similar to that of patients who also did not reach target LDL levels, thus suggesting that by achieving the goal LDL levels it would likely reduce the number of patients at risk for recurring atherothrombotic cardiovascular events. This relation is also supported by the distribution of the SMART Score risk classification by the Kaasenbrood et al. study (see Table 4) where the groups with highest risk amount to 66%, similar to the percentage of patients who did not reach LDL goals (see Table 5).

In the Dorresteijn et al. study, 42% of their population belonged to the three highest risk groups [9], as opposed to the present study which found a much higher percentage of these three groups with a total of 66.2% (see Table 3), this suggests that our population may have an increased residual cardiovascular risk profile. Similarly, the two highest risk groups of the Kaasenbrood study which was a 40% of the study sample, is significantly lower than the 66% we observed in the present study (see Table 4).

The EUROASPIRE study [15] tested whether the objectives formulated by the European Society of Cardiology in their 2016 European Guidelines on cardiovascular disease prevention were being met. This study concluded that in 2021, 71% of European patients under secondary prevention showed LDL-C levels of >70 mg/dL (greater than the current goal of <55 mg/dL), among which 84% were on medication to lower blood lipids, of which 60% were intensive therapy and 2.6% in combination [15,20].

More recent studies, such as the DA Vinci study [21], show data relating to control objectives according to the 2019 European Prevention Guidelines. In this study, only 18% of participants with pre-existing cardiovascular disease had LDL-C levels <55 mg/dL, and an underuse of intensive lipid-lowering therapies.

In our study, we found that only 35.2% of participants with CCS achieved LDL-C levels <55 mg/dL, which although higher than those in previously mentioned studies, is still far from the ideal. Additionally, it was found that 27.6% of participants were not on high intensity statin therapy despite having pre-existing cardiovascular disease, and underuse of combination therapy despite being known that cLDL levels is the factor that most contributes to residual risk. Statin monotherapy, even in high intensity regimens, does not alone help achieve goal levels of cLDL in the majority of patients, therefore combined therapies are necessary [[15], [20], [21], [22]].

Our results show similarly to those reported by Presta et al. [23] in Italy, that despite finding a population with a high prescription rate of high intensity statin therapies at 72.4%, there is an unexpected poor achievement of cLDL targets. In contrast, another study from Germany [24] that also assessed cLDL targets, reached only by 30% of their population, current statin therapy was low at 35%, compared to our report of 84.1% (see Table 9, Table 10).

#### 1.2.4 Conclusions

Data from the present study and complimentary studies referenced herein show that there is an inadequate control of cLDL levels in patients with CCS, and the appropriate available therapeutic resources aren't being utilized. Therefore, it is necessary to reflect upon the possible causes that give rise to this situation, such as therapeutic inertia, lack of secondary prevention programs and cardiac rehabilitation, as well as guidelines for continual care.

It is important to achieve a proper control of lipid levels in order to improve cardiovascular outcomes, despite currently being far from these goals. However, it may be possible to improve these metrics using intensive or combined lipid-lowering therapies more frequently, by ensuring a continued protocolled approach to care, and in a near future adding new pharmacologic options to current guidelines.

## Credit author statement

**Ginner O. Rizo-Rivera:** Conceptualization, Methodology, Resources, Formal Analysis Investigation, Data Curation, Writing – Original Draft, Supervision, Project Administration**; Marion Jose Valladares:** Formal analysis, Data curation, Writing – Review and Editing**; Hildebrando M. Toledo-Vargas:** Conceptualization, Methodology, Investigation, Resources**; Elibeth Chavez, Alejandro A. Garcia de la Rocha:** Conceptualization, Methodology, Investigation, Resources**; Luis A. Urcuyo-Hernandez:** Conceptualization, Methodology, Investigation, Resources**; Jose D. Meneses-Mercado:** Conceptualization, Methodology, Investigation, Resources**.**

## Funding disclosure

Self-funded

## Declaration of competing interest

No conflicts of interest to declare.
